# Characteristics of hepatic solitary necrotic nodules on contrast-enhanced ultrasonography

**DOI:** 10.1186/s12876-021-01608-9

**Published:** 2021-01-25

**Authors:** Chunyu Lu, Shaoshan Tang, Xiaoyue Zhang, Yang Wang, Kaiming Wang, Peng Shen

**Affiliations:** Department of Ultrasound, Shengjing Hospital of China Medical University, 36 Sanhao St, Heping District, Shenyang, 110004 Liaoning Province China

**Keywords:** Solitary necrotic nodules of liver, Ultrasonography, Contrast agent, Diagnosis, Differentiation, Contrast enhanced ultrasound

## Abstract

**Background:**

To summarize the characteristics of solitary necrotic nodules (SNN) in the liver observed under contrast-enhanced ultrasonography (CEUS).

**Methods:**

Conventional ultrasonography (US) and CEUS were performed in 24 patients who were confirmed to have SNN by pathological assessment. The US data and dynamic enhancement patterns of CEUS were recorded and retrospectively analyzed.

**Results:**

Ten of 24 patients underwent surgical resection, while the other 14 patients underwent a puncture biopsy to be confirmed as SNN. Among the 24 patients, 13 patients had a single lesion and 11 patients had multiple lesions. The largest lesion was selected for CEUS examination for patients with multiple lesions. Eleven patients presented no enhancement in all three phases, while the other 13 patients presented with a peripheral thin rim-like enhancement in the arterial phase, an iso-enhancement in the portal phase and delayed phase. However, no enhancement in the interior of the lesions was detected during three phases of CEUS.

**Conclusions:**

SNN has characteristic findings on the CEUS, which play an important role in the differential diagnoses of liver focal lesions.

## Background

Solitary necrotic nodules (SNN) of the liver are rare non-neoplastic nodular lesions which was first reported by Shepherd in 1983[1]. Until now, the pathogenesis of SNN was not clear, with several theories in the literature as follows [2, 3]: (1) central necrosis of hepatic hemangioma after sclerosis; (2) formation and progression of certain benign lesions after trauma in the liver; (3) parasitic infection; (4) malignant tumor of the digestive tract at the same time, which may lead to coagulative necrosis of liver due to allergic reaction. The clinical symptoms are not obvious, and it is difficult accurately diagnose SNN through conventional ultrasound (US) [4]. Most patients undergo enhanced computed tomography (CT) or enhanced magnetic resonance imaging (MRI) for diagnosis. With the wide application of contrast-enhanced ultrasonography (CEUS) in the diagnosis of liver lesions, some hepatic lesions could be qualitatively diagnosed by the enhancement pattern observed under CEUS [5]. This could be helpful to improve the differential diagnosis of hepatic lesions by comparing the difference in blood perfusion between the lesion and its surrounding normal liver parenchyma. In addition, CEUS has the advantage of displaying the lesion’s micro-vessels, which is helpful for the preoperative diagnosis of SNN, and avoids unnecessary puncture biopsy or surgical trauma. Since few studies have identified features of SNN on the CEUS for a differential diagnosis. In this study, we aimed to summarize the characteristics of hepatic SNN on CEUS and improve the diagnostic accuracy.

## Methods

### Patients

The ethics committee of our hospital approved this retrospective study and informed consent was obtained from all patients. From December 2010 to May 2020, 24 patients diagnosed with SNN by pathological results (surgical resection or puncture biopsy) in our hospital were analyzed retrospectively. This study included 6 males and 18 females, aged 24 to 67 years old, with an average of 50.33 ± 11.90 years. Most of the patients had no obvious clinical symptoms. Fifteen cases were examined by physical examination or hospitalized for other extrahepatic diseases, 2 patients were complicated with abdominal distension (abdominal pain or emaciation because of their gallbladder adenomyosis and gastritis), and 7 patients had a history of malignant tumor (2 cases of breast cancer, 3 cases of colon cancer and 2 cases of hepatocellular carcinoma). No patients had undergone any therapy such as interventional ablation or medicinal treatment before CEUS. All patients had Child–Pugh score A, 6 patients had fatty liver, 1 patient had hepatitis B with alpha fetal protein (AFP) elevation, 1 patient had hepatitis B alone, 3 patients had elevated carcinoembryonic antigen (CEA), and the remaining patients had no history of hepatitis, AFP, CEA, carbohydrate antigen 19–9 and 12–5 levels were normal (Table 1).

**Table 1:** General information of the patients [case (%)]

Project	Result
*Gender*	
Male	6 (25.00)
Female	18 (75.00)
*Examination reason*	
History of malignant	7 (29.17)
Clinical symptoms	2 (8.33)
Physical examination	15 (62.50)
*Disease background*	
Fatty liver	6 (25.00)
Hepatitis/cirrhosis	2 (8.33)
*Tumor biomarkers increase*	
AFP	1 (4.17)
CEA	3 (12.50)

### Examination acquisition

US and CEUS examinations were performed using SuperSonic Imagine (Aixplorer ultrasound system, France) or Toshiba Aplio500 (Toshiba Medical Systems, Tokyo, Japan), the central frequency of probe was 3.5–5 MHz, and the CEUS mechanical index was 0.06–0.15. The ultrasound contrast agent was Sonovue (diameter 2.5 μm produced by Bracco Company of Italy), and the main component was sulfur hexafluoride (SF_6_).

### US and CEUS

Conventional US was performed for all patients. Data including number, location, size, boundary, shape, internal echo of liver lesions and color Doppler images were recorded. CEUS was performed by injecting 2.4 ml of contrast agent through the anterior cubital vein, followed by 5 ml of saline solution. The dynamic perfusion process of the contrast agent and enhancement pattern of the lesions were observed and recorded on a hard disk for at least 5 min. The CEUS images were analyzed independently by two doctors (with 15 and 5 years of experience in CEUS imaging).

### Statistical analysis

All statistical analyses were performed with the SPSS version 19.0 software package (SPSS Inc, Chicago, IL), and the quantitative data which were normally distributed are expressed as mean ± standard deviation ($\overline{x}$ ± *s*). The differences in the lesion size between the different enhancement patterns on the CEUS were compared by an independent-samples *t* test. The differences in the lesion shape between the different enhancement patterns on the CEUS were compared by a Fisher's exact test. A *P* value less than 0.05 was considered statistically significant.

## Results

### Patient diagnosis and outcomes

All the patients were confirmed as SNN by pathological results, with 10 patients undergoing surgical resection and 14 patients undergoing a puncture biopsy. In the 14 patients with biopsy, the lesions were significantly reduced in 5 patients, and no significant changes were observed in the other 9 patients. One patient relapsed 4 years after surgical resection.

### Conventional ultrasound examination

Among the 24 patients, 13 patients had a single lesion and 11 patients had multiple lesions (six patients with 2 lesions,4 patients with 3 lesions, 1 patient with 4 lesions). There were 41 lesions in total. The largest lesion was selected for CEUS examination. Among the observed 24 lesions, 19 lesions were located in the right lobe while 5 lesions were located in the left lobe of the liver. Eleven cases had lesions close to the hepatic capsule (the distance between the edge of the lesion and the hepatic capsule was less than 1 cm). The maximum diameter of the lesions was 2.41 ± 0.88 cm (1.1–4.1 cm). The boundary of the lesions was clear in 19 cases and unclear in 5 cases. The lesions were regularly- round or oval-shaped in 16 cases, claw-like in 2 cases, bead-like in 1 case, dumbbell-like in 3 cases and nodular-like in 2 cases. The lesions were hypo-echoic in 18 cases, hyper-echoic in 2 cases, and heterogeneous mixed in 4 cases. There was no obvious blood flow signal in all lesions, and normal hepatic blood vessels could be seen around the lesions in 16 cases.

### Contrast-enhanced ultrasonography

**Fig. 1:**
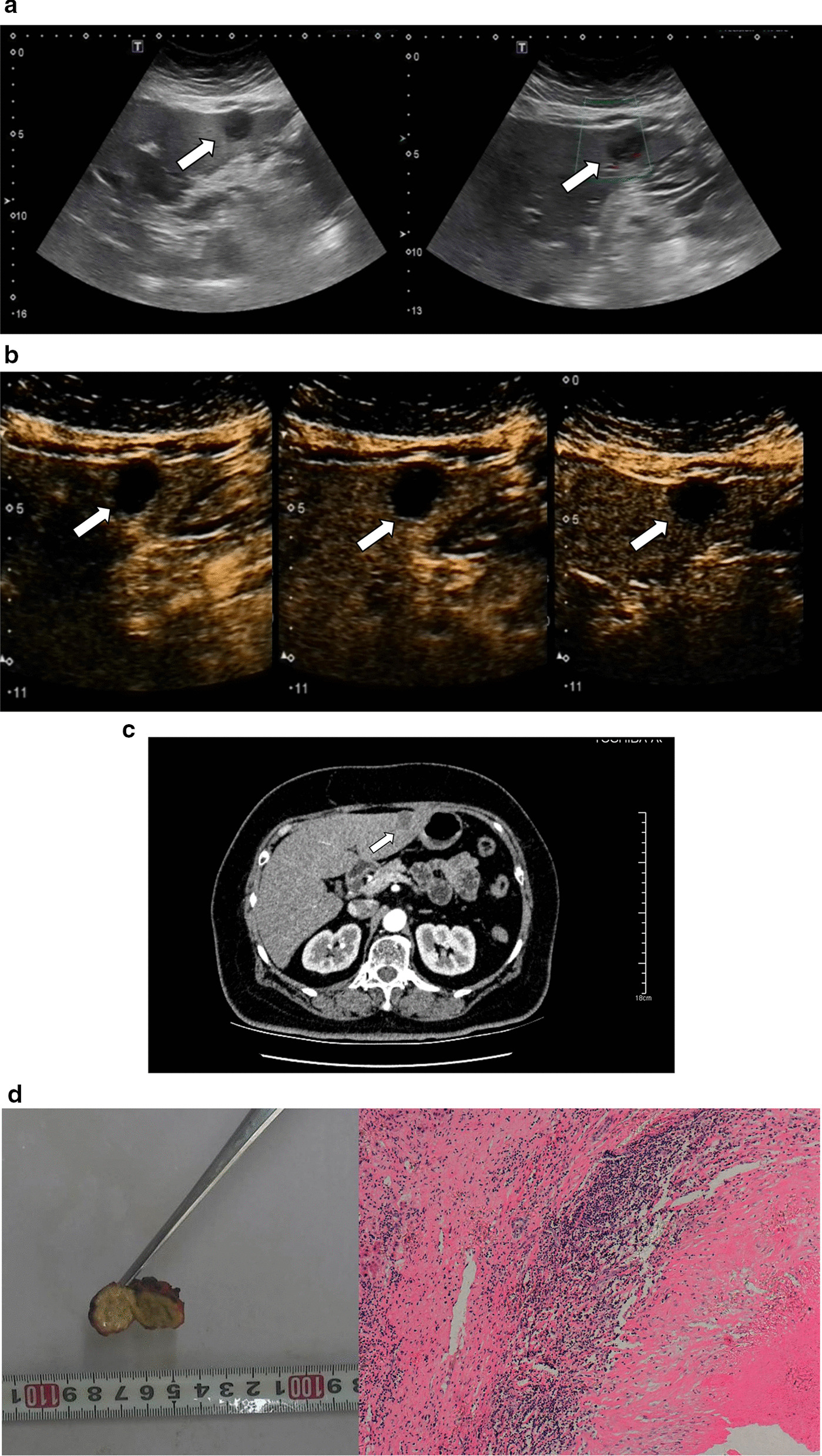
Images in a 60–70-year-old sex "2". **a** There was a hypo-echoic lesion under the hepatic capsule in segment 3 of the liver with a size of 2.2 cm × 2.1 cm. No obvious blood flow signal was detected in the lesion. **b** No contrast agent filling was found in the arterial phase (timer, 00: 24), portal phase (timer, 01:47), and delayed phase (timer, 02: 26). There was no enhancement in all the three phases. **c** Enhanced CT showed no obvious enhancement in the lesions. **d** The resected section of the specimen showed that the lesion was yellow- white and the boundary was clear. A large area of necrotic tissue with surrounding focal inflammatory cell infiltration could be seen (hematoxylin and eosin, × 100)

**Fig. 2:**
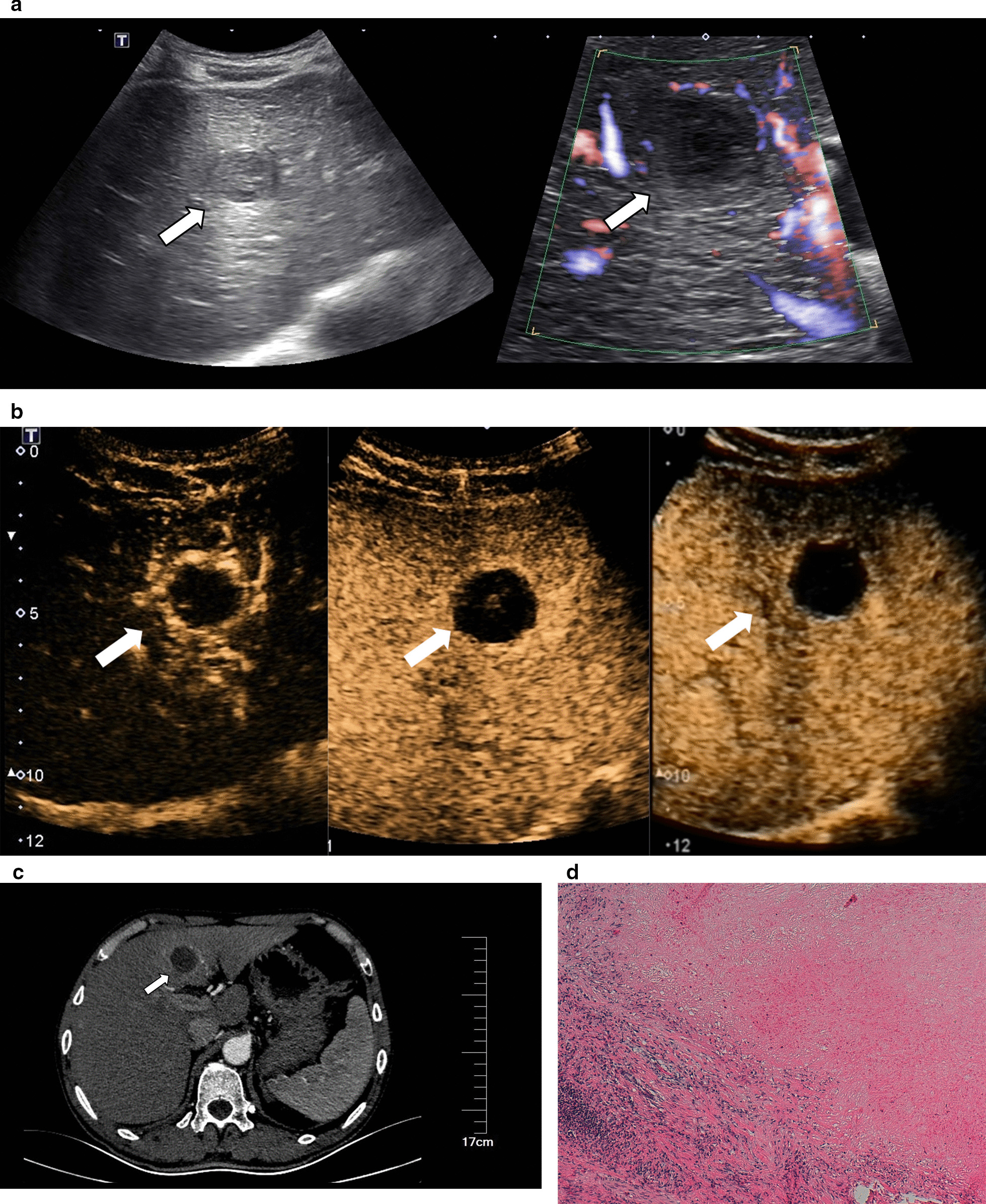
Images in a 50–60-year-old sex "1" with a history of hepatocellular carcinoma 3 years after surgery. **a** There was a hypo-echoic lesion in segment 4 of the liver with a size of 3.0 cm × 2.5 cm. Normal hepatic blood vessels could be seen around the lesions but no obvious blood flow signal was detected. **b** In arterial phase (timer, 00: 13), the lesions showed obvious thin peripheral rim-like enhancement, and the thickness of the enhanced ring was 0.36 cm; this demonstrated iso-enhancement in the portal phase (timer, 01: 29) and delayed phase (timer, 04:10). There was no contrast agent filling in the interior of the lesions. **c** Enhanced computed tomography showed no obvious enhancement in the lesions but enhancement in the boundary of the lesion. **d** Necrotic tissue of the surrounding liver tissue and focal inflammatory cell infiltration could be seen (hematoxylin and eosin, × 100)

**Fig. 3:**
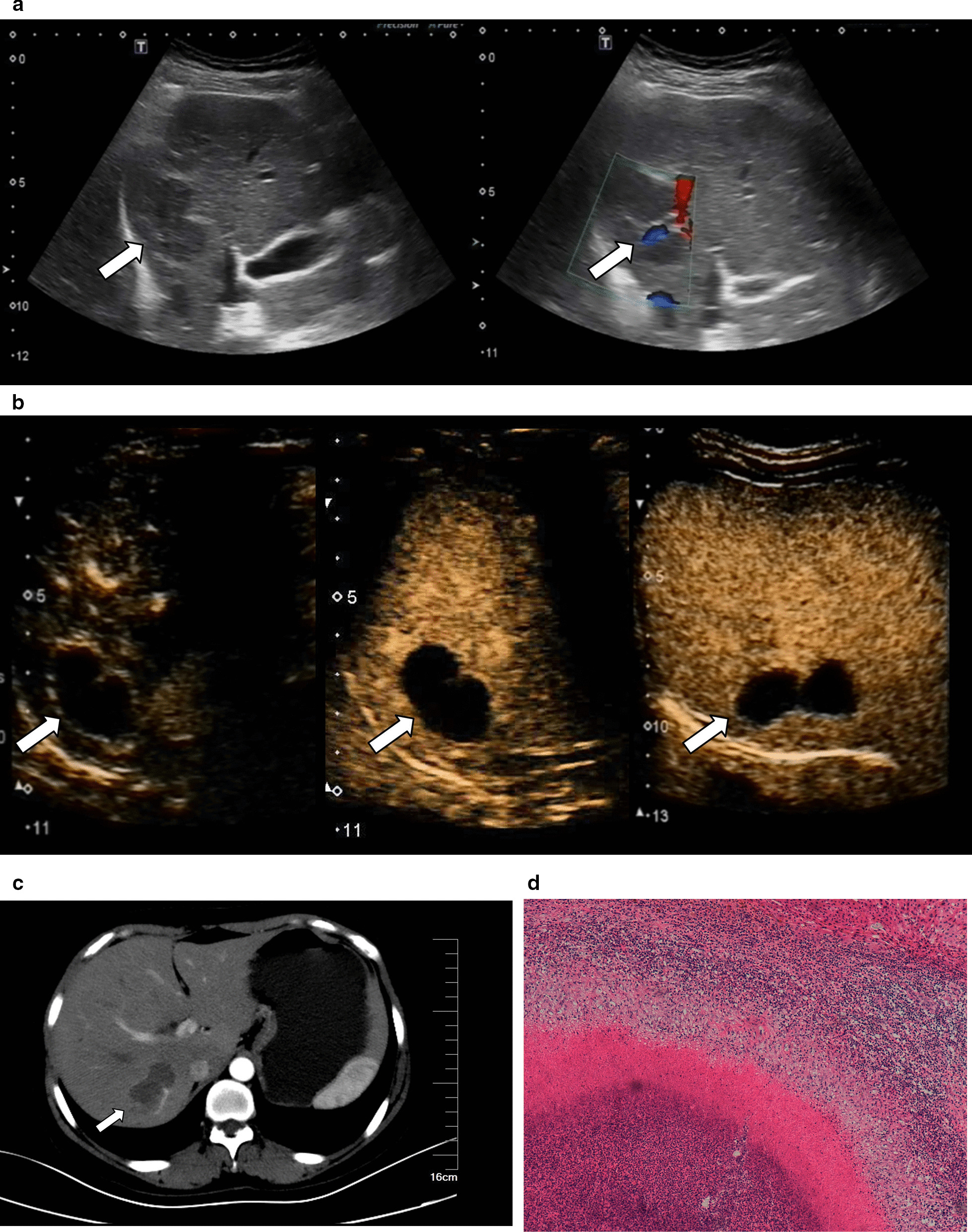
Images in a 40–50-year-old sex "2". **a** There were two hypo-echoic lesions in segment 7 of the liver which connected like a dumbbell. Normal hepatic blood vessels could be seen inside (the lesions connected portion) and around the lesions. **b** In the arterial phase (timer, 00:24), the lesions showed thin peripheral rim-like enhancement, and the thickness of the enhanced ring was 0.23 cm; this showed iso-enhancement in the portal phase (timer, 00: 57) and delayed phase (timer, 02: 03). There was no contrast agent filling in the interior of the lesions. **c** Enhanced CT showed no obvious enhancement in the lesions but showed enhanced blood vessels. **d** A large area of necrotic tissue with surrounding inflammatory cell infiltration could be seen (hematoxylin and eosin, × 100)

**Fig. 4:**
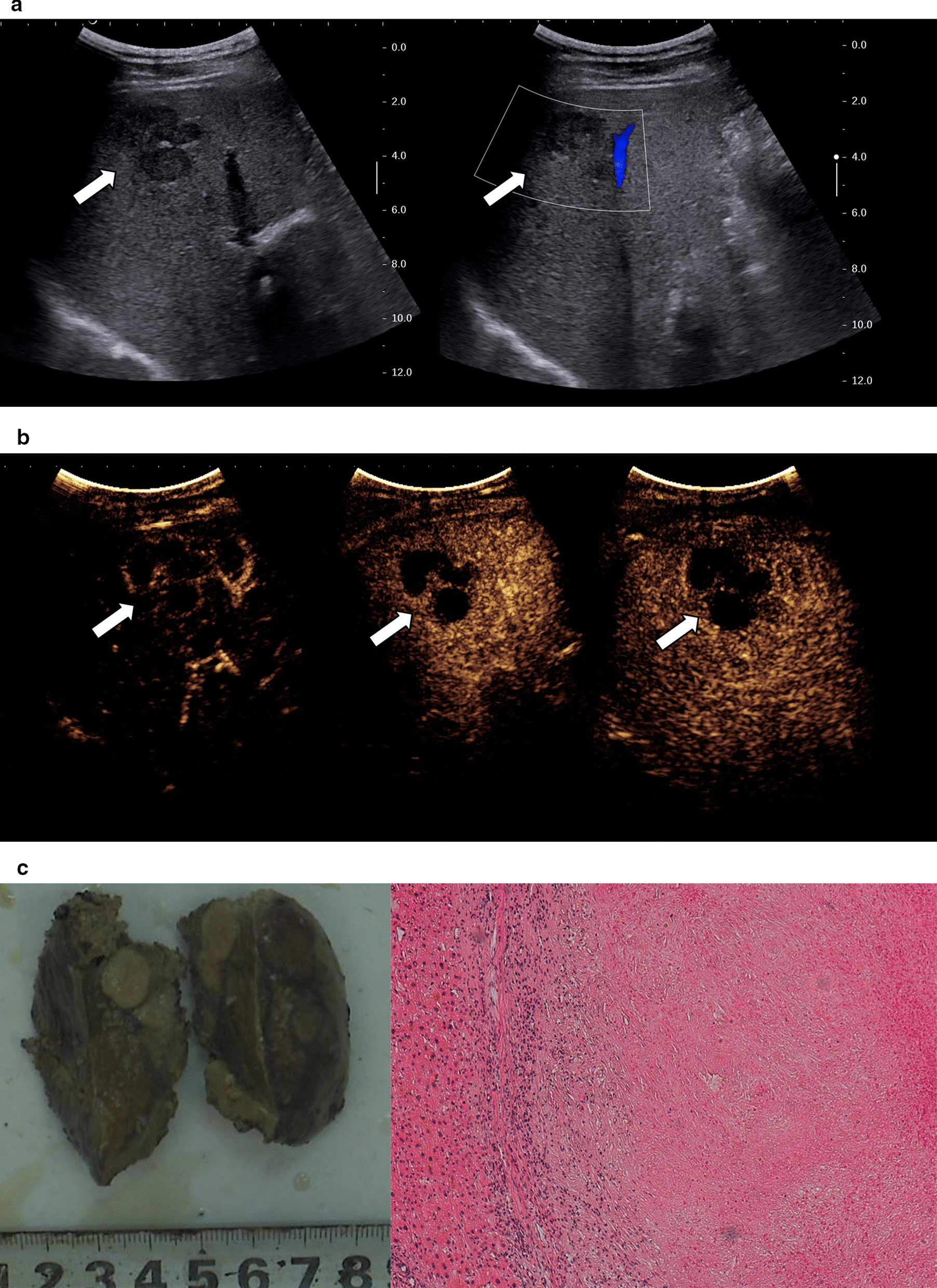
Images in a 50–60-year-old sex "1". a. Several hypo-echoic lesions were fused in segment 5 of the liver, which presented as a claw-like shape, with a size of approximately 3.8 cm × 2.8 cm. Color Doppler did not detect obvious blood flow signal in the lesions, and normal hepatic blood vessels could be seen in the surrounding region. b. In arterial phase (timer, 00:20), the lesions showed thin peripheral rim-like enhancement and septum enhancement in the interior lesions, but most of the lesions were not enhanced, and the thickness of the enhanced ring around the lesions was 0.21–0.25 cm. The enhanced portion of the lesions showed iso-enhancement in the portal phase (timer, 00:40) and delayed phase (timer, 02:05), and the center of the lesions were not enhanced in these two phases. c. On the section of the resected specimen, the lesions were yellow white, with focal lesions fusing, and the boundary was clear. The lesions were necrotic tissue, and hyperplastic fibrous tissue and inflammatory cell were seen around the lesions(hematoxylin and eosin, × 100)

The CEUS enhancement pattern was divided into 2 types. Type I was no enhancement in all three phases compared with the surrounding liver parenchyma (Fig. 1): 11 cases belonged to type I. Type II was presence of peripheral thin rim-like hyper-enhancement in the arterial phase and iso-enhancement in the portal phase and delayed phases, with no enhancement in the interior of the lesions during all three phases (Figs. 2, 3, 4): 13 cases showed a type II enhancement pattern. The non-enhanced area remained unchanged during three phases (Table 2). The boundary of the enhancement ring was clear and sharp. The thickness of the enhancement ring was 0.30 ± 0.15 cm. There was a significant difference in lesion size between the two enhancement patterns (*P* = 0.005 < 0.05). The lesions of type II (2.83 ± 0.87 cm) were larger than lesions of type I (1.92 ± 0.61 cm). There was no significant difference in lesion shape between the two enhancement patterns (*P* = 0.211 > 0.05).

**Table 2.** 24 patients of SNN US and CEUS imaging [case (%)]

Project	Result
*Single/multiple lesions*	
Single lesion	13(54.17)
Multiple lesions	11(45.83)
*Lesion site*	
Left lobe	5(20.83)
Right lobe	19(79.17)
*Distance between lesion and hepatic capsule*	
> 1 cm	13(54.17)
< 1 cm	11(45.83)
*Lesion morphology*	
Regularity	16(66.67)
Claw-like	2(8.33)
Bead-like	1(4.17)
Nodular-like	2(8.33)
Dumbbell-like	3(12.50)
*Echo of lesion*	
Hypo-echoic	18(75.00)
Hyper-echoic	2(8.33)
Heterogeneous echoic	4(16.67)
*Lesion boundary*	
Clear	19(79.17)
Unclear	5(20.83)
*Normal hepatic blood vessels in the periphery*	
Yes	16(66.67)
No	8(33.33)
*Enhancement pattern*	
No enhancement	11(45.83)
thin rim-like enhancement	13(54.17)

### Pathology

The resected section of the specimen was yellow or white, and some of them were gray-white or grayish yellow, with or without a thin capsule, and the boundary was clear. The textures of the lesions were slightly hard and partially fused. Hematoxylin and eosin (HE) staining showed that the lesions were mainly homogeneous coagulation necrotic tissue, surrounded by proliferated fibrous tissue, with surrounding focal infiltration of inflammatory cells and multinucleated giant cells, and punctate necrosis surrounding the liver tissue.

## Discussion

SNN is a rare non-neoplastic lesion of the liver which often occurs in patients aged between 60 to 70 years, but it had also been reported to occur between the ages of 30 to 40 years, with more males being affected than females [6]. Most patients showed no clinical symptoms, and were detected occasionally during physical examination, postmortem examination, or operation [7]. The pathological features of SNN include nodular coagulation necrosis, and presence of a complete fibrous capsule around the lesion, with the infiltration of inflammatory cells in the capsule (mainly eosinophil cells and lymphocytes), and no vascular tissue in the lesion [4, 8].

It had been reported that the SNN lesions could reduce or disappear after conservative treatment. So, if SNN is highly suspected, surgical resection should be avoided [9, 10]. Therefore, a correct diagnosis before the operation is crucial.

There are no specific clinical symptoms and laboratory tests for the diagnosis of SNN [4, 11]. SNN often presents as hypo-echoic nodules on the conventional US, which lacks specificity, and thus makes it difficult to make a qualitative diagnosis. [12–14]. Color Doppler US has some limitations in the detection of the micro-vessels in the lesions, and puncture biopsy or surgical resection are the ultimate diagnostic methods and gold standard for SNN detection [15]. With the development of CEUS, the process of internal and peripheral blood perfusion can be dynamically observed in real time, which could effectively improve the accuracy of SNN diagnosis and reduce unnecessary biopsy or surgical trauma [16, 17].

In this study, 11 patients (11/24) showed no enhancement in the three phases, which was similar to the report by Wang et al.[18]. However, in our study, 13 patients (13/24) showed peripheral thin rim-like enhancement in the arterial phase, which could easily be misdiagnosed as a malignant tumor such as metastatic hepatic carcinoma (MHC). However, the inner wall of the lesion’s enhancement ring was clear and sharp, and demonstrated iso-enhancement in the portal and delayed phases, which differed from the obviously decreased enhancement ring of the MHC lesion in the portal phase [2, 8]. Peripheral thin rim-like enhancement in the arterial phase suggested micro-vessel distribution around the lesions of the SNN. HE staining showed that the edges of the lesions were hyperplastic fibrous tissue and inflammatory response bands caused by infiltrated inflammatory cells. This might be the pathological basis of the peripheral thin rim-like enhancement observed in the arterial phase [4]. Some lesions showed septum-like enhancement, which might be related to fibrous tissue separation following multi-lesion fusion. Internal septum-like enhancement may be one of the characteristics of multi-lesion fusion. There was a significant difference in lesion size between the two enhancement patterns: the lesions which showed a peripheral thin rim-like enhancement were larger than those without enhancement. This suggested that the lesions were in different pathologic stage: those without enhancement might be at the end-stage and tended to atrophy. The lesions with peripheral thin rim-like enhancement might be at the relatively early stage and maintain the peripheral vessel distribution.

Previous literature has shown that presence of a marked peripheral rim-like enhancement with internal hypo-intensity on a longer MRI (a delayed time of 1–2 h) was helpful in the diagnosis of SNN [19]. The contrast agent used during an MRI is different from that used during a CEUS. The ultrasonic contrast agent is a blood-pool imaging agent, which does not diffuse to the intercellular space and does not show delayed enhancement. The MRI contrast agent is an extracellular contrast agent, which can diffuse to the extracellular space. Because the contrast medium diffuses slowly between blood vessels and the fibrous tissue, the peripheral rim-like enhancement of the lesions is observed 1–2 h after injection of the contrast agent. The interval is relatively long, making it unsuitable for routine clinical examination. SNN shows specific manifestations at the early stage of CEUS, which could reduce the examination time and gave better repeatability, thus making CEUS more suitable for clinical use.

SNN with peripheral rim-like enhancement in the arterial phase should especially be distinguished from MHC. Previous studies had reported that when MHC lesions appeared to be necrotic, it could present peripheral rim-like enhancement in the arterial phase with the interior of the lesion showing no enhancement in the three phases [20–22]. However, there was a significant difference in the thickness of the enhancement ring in the arterial phase between MHC and SNN. The enhancement rings of the MHC were thicker than that of the SNN [23]. Besides, the enhancement ring of the MHC was infiltrated by malignant tissue, and the contrast agent was washed out during the portal phase and demonstrated hypo-enhancement. Therefore, the hypoechoic area became larger in the delayed phase. However, the enhancement ring of the SNN was a thin fibrous capsule without obviously being washed out in the delayed phase; therefore, the hypoechoic area showed no significant change compared the arterial phase [24].

The peripheral rim-like enhancement observed on the CEUS can also be detected in some cases of intrahepatic cholangiocarcinoma (ICC). However, the enhancement ring in ICC is unevenly thicker with an irregular shape, and the interior of the lesion has a contrast agent filling. Clinical characteristics and specific laboratory examinations are also helpful for the differential diagnosis of SNN [25–27].

When an abscess with liquefied and necrotic tissue is formed, a peripheral rim-like enhancement is observed in the arterial phase of CEUS, but honeycomb enhancement of the internal part can help for differentiation [28–30].

Hepatic alveolar echinococcosis (HAE) is also a rare parasitic disease with a long incubation period and lack of specific clinical symptoms [31]. There is no contrast media filling in lesions during all the three phases in HAE. The combination of contact history in the epidemic area and specific laboratory examination could be helpful for a differential diagnosis [32, 33].

SNN needs to be differentiated from hypovascular hepatocellular carcinoma (HCC). Although more than 90% of the HCC are hypervascular, few of HCC may showing little or no obvious enhancement in the inner of the lesion on CEUS, but there is no obvious rim enhancement around the lesion. It is difficult to differentiate HCC from SNN to those lesions without obvious enhancement. Puncture biopsy can be performed when it is difficult to differentiate HCC from SNN. Hepatitis or liver cirrhosis background and the increase of AFP are also helpful to the differential diagnosis [34, 35].

One case of this study relapsed 4 years after surgery, which has not been previously reported. The lesions showed no enhancement in all three phases during both CEUS examinations between four years. Therefore, although SNN is a benign lesion, there is a possibility of recurrence. Such patients who choose conservative treatment require attention and should be closely followed up.

This study was the first to report two types of enhancement patterns observed in SNN, and a case of relapse, which has not been reported in the literature. However, some limitations should also be noted, as this study was a retrospective study and with a relatively small number of cases. A larger sample size and prospective study is required for further investigation.

## Conclusions

In conclusion, SNN has specific characteristic findings on the CEUS, which can improve the diagnostic accuracy and CEUS can be an effective technique for the diagnosis of SNN.

## Data Availability

The raw data generated and analyzed in the current study are not publicly available due to appropriate protection of patient personal information but are available from the corresponding author on reasonable request.

## Abbreviations

AFP: Alpha fetal protein
CEA: Carcinoembryonic antigen
CEUS: Contrast-enhanced ultrasonography
CT: Computed tomography
HAE: Hepatic alveolar echinococcosis
HCC: Hepatocellular carcinoma
HE: Hematoxylin and eosin
ICC: Intrahepatic cholangiocarcinoma
IPL: Inflammatory pseudo-tumor of the liver
MHC: Metastatic hepatic carcinoma,
MRI: Magnetic resonance imaging,
SNN: Solitary necrotic nodules
US: Conventional ultrasound
